# Prevalence of Ventilator-Associated Pneumonia in Neonates in a Tertiary Care Hospital in Western Nepal

**DOI:** 10.31729/jnma.4295

**Published:** 2019-04-30

**Authors:** Anita Lamichhane, Aparna Mishra

**Affiliations:** 1 Department of Pediatrics, Lumbini Medical College, Palpa, Nepal

**Keywords:** *mechanical ventilation*, *neonatal intensive care unit*, *ventilator-associated pneumonia*

## Abstract

**Introduction:**

Ventilator-associated pneumonia is a serious problem which needs to be addressed for a better outcome of the ventilated babies. The present study is undertaken to find out the prevalence of ventilator-associated pneumonia in neonates in a tertiary care hospital in western Nepal.

**Methods:**

A descriptive cross-sectional study was carried out in a tertiary care hospital in the western region of Nepal from March 2016 to February 2019 after approval from the Institutional review committee. Sample size was calculated and convenience sampling was done to reach the sample size. Data were collected from hospital records and entered in Statistical Package for the Social Sciences, point estimate at 95% confidence interval was calculated along with frequency and proportion for binary data.

**Results:**

Out of 95 patients ventilated in neonatal intensive care unit in the last 3 years, 23 (24.01%) developed ventilator-associated pneumonia. Prevalence of ventilator-associated pneumonia is 23 (24.01%) at 95% (14%-34%). Late onset ventilator associated pneumonia was seen in 15 (15.78%) while early onset ventilator associated pneumonia was seen in 8 (8.42%).

**Conclusions:**

Prevalence of ventilator associated pneumonia in neonates in tertiary care hospital is high compared to other studies conducted in neonates.

## INTRODUCTION

Ventilator-associated pneumonia (VAP) is a serious complication in neonates on mechanical ventilation and accounts for 6.8–32.2% of healthcare-associated infections among neonates.^1^ VAP is the nosocomial pneumonia in mechanically ventilated patients developing>48 hours after initiation of mechanical ventilation and was neither present nor incubating at the time of intubation.^2^ VAP can be early onset (< 96 hours) or late onset (>96 hours).^3^ It is associated with complications especially when the ventilator care is prolonged, in intensive care units of developing countries.^2^

Despite important geographical variations, there are certain groups of organisms which constitute for more than 80% of VAP.^4^ The management of children with invasive ventilation in developing countries with limited resources is challenging. There is a paucity of data of the neonates undergoing mechanical ventilation and its various complications in our country.

The aim of the study was to find the prevalence of ventilator-associated pneumonia in neonates in a tertiary care hospital in western Nepal.

## METHODS

This descriptive cross-sectional study was conducted in the Neonatal Intensive Care Unit (NICU) of a tertiary care institute, Lumbini Medical College, Palpa of Western Nepal, from March 2016 to Feb 2019 after approval from the Institutional Review Committee (IRC) of the college. Study population is mechanical ventilated neonates who were admitted in the hospital. The present study included all the inborn and outborn ventilated neonates (<28 days) for more than 12 hours and not having suspected metabolic disorders. The present study did not include those neonates requiring mechanical ventilation for less than 12 hours, babies who have undergone recent surgical interventions and neonates with congenital anomalies like Down's syndrome.

All the information including birth weight, gestational age at the time of delivery, sex, mode and place of delivery, clinical signs and symptoms, indication for ventilation, maternal risk factors, development of pneumonia while on ventilator was collected from the manual search of case files with permission from the concerned authority.

Convenience sampling was done and sample size was calculated using the formula,


$$\begin{array}{lll} \text{n} & = & \frac{{\text{Z}}^{2}\times \text{p}\times \text{q}}{{\text{d}}^{2}}\\ & = & {\text{(1}.96)}^{2}\times 0.45\times \text{(1-0}.45)/(0{.1)}^{2}\\ & = & 95 \end{array}$$

Where,

Z = 1.96 at 95% CI.
p= prevalence of VAP (educated guess), 45%
q= 1-p
d= margin of error, 10% Total sample size was calculated to be 95.

Selection bias and information bias has been minimized as possible. Data were collected from hospital records and entered in a SPSS, point estimate at 95% CI was calculated along with frequency and proportion for binary data.

## RESULTS

During the study period, out of 95 patients ventilated in neonatal intensive care unit, 23 (24.21 %) patients developed VAP. Prevalence of ventilator-associated pneumonia is 23 (24.01%) at 95% CI (14%-34%). Early-onset VAP (<96 hrs) was observed in 8 (8.42%) babies while late-onset VAP (>96 hrs) developed in 15 (15.79%) patients.

The most common indication for ventilation was sepsis 34 (35.79%) followed by respiratory distress 15 (15.9%) (Table 1).

**Table 1:** Showing indications for ventilation in neonates.

Cause	n (% )
Apnoea	3 (3.16 )
Aspiration pneumonia	2 (2.11)
Pneumonia	8 (8.42)
Respiratory distress	15 (15.79)
Meconium aspiration	6 (6.32)
Prematurity	9 (9.47)
Shock	6 (6.32)
Cardiac arrest	3 (3.16)
Congenital pneumonia	2 (2.11)
RDS with sepsis	1 (1.05)
Seizure	7 (7.37)
Sepsis	34 (35.79)

Demographic details of the babies are depicted (Table 2). Mean weight of the babies were 2.5±0.75 kg while the mean age was 3.94±6.5 days. Among the admitted neonates, 5 (5.26%) were <32 weeks, 24 (25.26%) were between 33–37 weeks, 64 (64.36%) were >37 weeks of gestation and 2 (2.10%) were beyond 42 weeks.

**Table 2:** Showing the demographic profile of the neonatal ventilated patients with VAP and without VAP.

	No of babies ventilated (n = 95)	
Characteristics	With VAP (n = 23) n (%)	Without VAP (n = 72) n (%)
Place of birth		
Inborn	19 (82.60)	58 (80.56)
Outborn	4 (17.40)	14 (19.44)
Mode of delivery		
Vaginal	10 (43.48)	43 (59.72)
Caesarean	12 (52.18)	24 (33.33)
Vacuum	1 (4.35)	5 (6.94)
Gender		
Male	12 (52.18)	49 (69.06)
Female	11 (47.82)	23 (31.94)
Birth weight (g)		
1000–1500	2 (8.70)	6 (8.33)
1501–2000	3 (13.04)	22 (30.56)
2001–2500	13 (56.53)	16 (22.22)
>2500	5 (21.73)	28 (38.89)
Gestational age (weeks)		
<32	1 (4.35)	4 (5.56)
33–37	5 (21.73)	19 (26.39)
37–42	17 (73.92)	47 (65.28)
>42	0	2 (2.77)

## DISCUSSION

During the study period, 644 patients were admitted in NICU, of which 95 (14.75%) were mechanically ventilated. The prevalence of VAP was found out to be 24.21% in our study. Other studies have reported incidence of VAP ranging from low of 13.2%^5^ to a high of 50%. ^6^ Khattab A et al. reported ventilator associated pneumonia in 55.2% neonates on mechanical ventilator.^7^ A study done by Bozorgmehr R et al. concluded with prevalence of VAP as 11%.^8^ In one of the studies conducted in Germany, the incidence was only 2.2%.^9^ Sepsis was the most common reason, 35.79% for ventilation followed by respiratory distress, 15.79% in contrary to a study^10^ done at Patan hospital, Nepal which showed 33% HMD and 24% severe sepsis as indications for ventilation. The commonest reason for ventilation was respiratory distress 63% in West Indies, birth asphyxia 60% in Paropakar Maternity and Women's Hospital, Nepal and 34% in BPKIHS, Nepal.^11–14^

The incidence of late-onset VAP was more common than early onset VAP. Similar results were observed in studies done by Tripathi et al. where late-onset VAP was more common than early onset VAP 53.3% versus 46.7% respectively. ^12^

## CONCLUSIONS

Prevalence of ventilator associated pneumonia in neonates in tertiary care hospital is high compared to other studies conducted in neonates.

## Conflict of Interest

**None.**

## References

[1] Sodhi MK, Kumar GAD, Singh K, Kumar A, Malhotra S, Neki NS. (2018). Incidence, Clinical and Microbiological Pattern of Ventilator Associated Pneumonia (VAP) In Neonatal Intensive Care Unit in Amritsar, India. Int J Curr Res Med Sci..

[2] Joseph N, Sistla S, Dutta T, Badhe A, Parija S. (2009). Ventilator-associated pneumonia in a tertiary care hospital in India: incidence and risk factors. J Infect Dev Ctries.

[3] Foglia E, Meier MD, Elward A. (2007). Ventilator-associated pneumonia in neonatal and pediatric intensive care unit patients. Clin Microbiol Rev..

[4] Sandiumenge A, Rello J. (2012). Ventilator-associated pneumonia caused by ESKAPE organisms: cause, clinical features, and management. Curr Opin Pulm Med..

[5] Lee PL, Lee WT, Chen HL. (2017). Ventilator- Associated Pneumonia in Low Birth Weight Neonates at a Neonatal Intensive Care Unit: A Retrospective Observational Study. Pediatrics and Neonatology.

[6] Malhotra P, Kumar N, Thapar K, Bagga AK. (2018). Comparative study of incidence, risk factors, etiological agents and outcome of early and late ventilator-associated pneumonia in pediatric intensive care unit at a tertiary care centre. Int J Contemp Pediatr..

[7] Khattab AA, El-Lahony DM, Soliman WF. (2014). Ventilator-associated pneumonia in NICU. Menoufia Med J..

[8] Bozorgmehr R, Bahrani V, Fatemi A. (2017). Ventilator-Associated Pneumonia and Its Responsible Germs; an Epidemiological Study. Emerg (Tehran).

[9] Leistner R, Piening B, Gastmeier P, Geffers C, Schwab F. (2013). Nosocomial infections in very low birthweight infants in Germany: current data from the National Surveillance System NEO-KISS. Klinische Padiatrie.

[10] Shrestha S, Karki U. (2012). Indicators of admission and outcome on a newly established neonatal intensive care unit in a developing country (Nepal). Nepal Med Coll J..

[11] Trotman H (2006). The Neonatal Intensive Care Unit at the University Hospital of the West Indies- The Shrestha first few years' experience. West Indian Med J..

[12] Tripathi S, Malik G.K, Jain A, Kohli N. (2010). Study of Ventilator-Associated Pneumonia in Neonatal Intensive Care Unit: characteristics risk factors and outcome. Internet J Medical Update.

[13] Modi P, Javadekar T, Nanda S, Pandya N. (2012). A study on ventilator-associated pneumonia in pediatric age group in a tertiary care Hospital, Vadodara. National Journal of Medical Research.

[14] Badr MA, Ali YF, Albanna EA, Basir MR, Amr GE. (2011). Ventilator-associated pneumonia in critically-ill neonates admitted to neonatal intensive care unit, Zagazig university hospitals. Iran J Pediatr..

